# Carboplatin in Patients With Metastatic Castration-Resistant Prostate Cancer Harboring Somatic or Germline Homologous Recombination Repair Gene Mutations: Phase II Single-Arm Trial

**DOI:** 10.2196/54086

**Published:** 2024-04-18

**Authors:** Rishabh Jain, Akash Kumar, Atul Sharma, Ranjit Kumar Sahoo, Aparna Sharma, Amlesh Seth, Brusabhanu Nayak, Shamim A Shamim, Seema Kaushal, Haresh KP, Chandan J Das, Atul Batra

**Affiliations:** 1 Department of Medical Oncology Dr BR Ambedkar Institute Rotary Cancer Hospital All India Institute of Medical Sciences New Delhi India; 2 Department of Urology All India Institute of Medical Sciences New Delhi India; 3 Department of Nuclear Medicine All India Institute of Medical Sciences New Delhi India; 4 Department of Pathology All India Institute of Medical Sciences New Delhi India; 5 Department of Radiation Oncology Dr BR Ambedkar Institute Rotary Cancer Hospital All India Institute of Medical Sciences New Delhi India; 6 Department of Radiology All India Institute of Medical Sciences New Delhi India; 7 Department of Medical Oncology New Delhi India

**Keywords:** carboplatin, mCRPC, prostate cancer, homologous recombinant gene repair, metastatic castration-resistant prostate cancer, incurable, deleterious mutation, synthetic lethality, tumor, DNA, low-income, middle-income, chemotherapeutic, drug, retrospective study, taxane, novel antiandrogen, single-arm study, health-related, quality of life, bone lesion

## Abstract

**Background:**

Approximately 20%-25% of patients with metastatic castration-resistant prostate cancer (mCRPC) harbor a deleterious germline or somatic mutation in the homologous recombination repair (HRR) pathway genes, which is involved in the repair of double-stranded DNA damage. Half of these mutations are germline, while the remaining are exclusively somatic. While polyadenosine 5’diphosphoribose [poly (ADP-ribose)] polymerase inhibitors, such as olaparib and rucaparib, are effective in this subgroup, their widespread use is limited due to the associated high cost, especially in resource-constrained settings. Notably, platinum agents like carboplatin have exquisite sensitivity to cells with defective DNA repair machinery. Carboplatin, a conventional, inexpensive chemotherapeutic agent, offers a potential alternative treatment in such patients. Several retrospective small case series support this hypothesis. However, there are no prospective clinical trials of carboplatin in patients with mCRPC with HRR mutations.

**Objective:**

The primary objective is to assess the objective response rate of 3 weekly carboplatin treatments in patients with mCRPC harboring deleterious mutations in the HRR pathway genes and previously treated with a taxane or a novel antiandrogen agent. The secondary objectives include progression-free survival, health-related quality of life, and safety profile of carboplatin.

**Methods:**

Patients diagnosed with mCRPC harboring HRR pathway mutations previously treated with docetaxel or novel antiandrogen agents (abiraterone, enzalutamide, apalutamide, or darolutamide) or both will be eligible. Genes involved directly or indirectly in the HRR pathway will be tested. In this single-arm phase II study, we will screen approximately 200 patients to enroll 49 patients, and carboplatin (dosing at the area under curve=5) will be administered every 3 weeks until progression or intolerable side effects. The primary end point will be assessed as the proportion of patients with a reduction of serum prostate-specific antigen by more than 50% from enrollment. Secondary outcomes include progression-free survival—soft-tissue disease progression (by response evaluation criteria in solid tumors, version 1.1, and bone lesion progression using Prostate Cancer Clinical Trials Working Group 3 criteria), health-related quality of life during carboplatin treatment using the Functional Assessment of Cancer Therapy—Prostate questionnaire and the European Organisation for Research and Treatment of Cancer questionnaire and safety profile of carboplatin (National Cancer Institute’s Common Terminology Criteria for Adverse Events version 5.0).

**Results:**

The trial started enrollment in September 2023. This trial is ongoing, and 12 patients have been recruited to date. All 49 participants will be enrolled according to plan.

**Conclusions:**

This prospective phase II trial represents a critical step toward addressing the therapeutic gap in patients with mCRPC harboring HRR pathway mutations, particularly in demographic regions with limited access to poly (ADP-ribose) polymerase inhibitors. Outcomes from this study will inform clinical practice and guide future phase III randomized trials, ultimately improving patient outcomes globally.

**Trial Registration:**

Clinical Trials Registry of India CTRI/2023/04/051507; https://ctri.nic.in/Clinicaltrials/pmaindet2.php?EncHid=Njc0NjU=&Enc=&userName=

**International Registered Report Identifier (IRRID):**

DERR1-10.2196/54086

## Introduction

Patients with metastatic prostate cancer are treated with androgen deprivation therapy, chemotherapeutic agents, or novel antiandrogen agents [1]. These patients eventually develop resistance to androgen deprivation therapy, and this state, known as castration-resistant prostate cancer, is incurable despite the advent of several newer therapies [2]. The therapeutic landscape for metastatic castration-resistant prostate cancer (mCRPC) includes chemotherapeutic agents, novel antiandrogen agents, and radioligand therapies [3].

Pritchard et al [4] reported that 11.8% of men with metastatic prostate cancer harbor deleterious germline mutations, primarily in *BRCA2*, followed by *ATM*, *CHEK2*, *BRCA1*, *RAD51D*, and *PALB41* genes. Subsequent studies evaluated the prevalence of deleterious somatic mutations within the homologous recombination repair (HRR) pathway in 25%-30% of patients with mCRPC [5]. Polyadenosine 5’diphosphoribose (poly [ADP-ribose]) polymerase inhibitor (PARP) inhibitors employ a strategy of synthetic lethality, inhibiting the base excision repair pathway, leading to the accumulation of unrepaired DNA breaks within HRR-deficient tumor cells, culminating in cancer cell death [6-8]. Olaparib, a PARP inhibitor, demonstrated an impressive 88% response rate in patients with mCRPC with HRR gene abnormalities, in contrast to a minimal 3% response in those without these mutations [9,10]. The pivotal PROfound trial supported these findings, leading to Food and Drug Administration approval for patients with mCRPC harboring HRR gene mutations [5]. Similar efficacy was observed with other PARP inhibitors, including rucaparib and talazoparib [11,12]. However, most patients with mCRPC harboring HRR gene mutations residing in low- and middle-income countries do not have access to PARP inhibitors due to the high cost.

Interestingly, HRR-deficient tumors also exhibit increased sensitivity to platinum chemotherapeutic agents (eg, carboplatin) due to their reliance on the HRR pathway for DNA double-strand break repair. This vulnerability has been exploited by platinum-containing treatments in breast and ovarian tumors [13,14]. Initial retrospective studies involving carboplatin in unselected patients with mCRPC showed encouraging efficacy, particularly within the subset carrying HRR gene alterations [15-17]. However, these studies are limited by several biases.

Despite a strong scientific rationale and encouraging preliminary data from real-world studies, no prospective study has been reported to assess the role of carboplatin in this subset of patients. Therefore, we planned this phase II single-arm study to evaluate the efficacy of carboplatin in patients with metastatic prostate cancer harboring mutations in the HRR pathway.

## Methods

### Objectives

This study is an investigator-initiated, prospective phase II, single-arm clinical trial. The primary objective of this study is to assess the prostate-specific antigen (PSA) response rate of 3 weekly carboplatin (dose at area under the curve 5) in patients with mCRPC harboring deleterious or likely deleterious mutations in the HRR genes and previously treated with a taxane or a novel antiandrogen (proportion of patients with more than 50% serum PSA decline).

The secondary objectives are to compare the effect of oral gabapentin with placebo on the following:

- Progression-free survival—soft-tissue disease progression (by response evaluation criteria in solid tumors, version 1.1) and bone lesion progression (by Prostate Cancer Clinical Trials Working Group 3 criteria)
- Health-related quality of life during carboplatin treatment (Functional Assessment of Cancer Therapy—Prostate questionnaire and European Organisation for Research and Treatment of Cancer questionnaire)
- The safety profile of carboplatin (National Cancer Institute’s Common Terminology Criteria for Adverse Events, version 5.0)

### Study Setting

This study is being conducted in the outpatient department of Dr BR Ambedkar Institute Rotary Cancer Hospital, New Delhi, India, and the National Cancer Institute, Jhajjar, the 2 cancer blocks of the All India Institute of Medical Sciences, New Delhi, a central government-funded tertiary care teaching hospital. The catchment area includes an over 20 million population from the Northern states of India, with approximately 15,000 patients with cancer being treated every year.

### Eligibility Criteria

All the following criteria must be met for enrollment:

- Histological diagnosis of prostate cancer.
- Serum testosterone <50 ng/dL within 28 days before screening.
- Documented current evidence of mCRPC, where metastatic status is defined as at least one documented metastatic lesion on either a bone scan, computed tomography, or magnetic resonance imaging scan.
- Prior treatment with docetaxel or at least one of the novel antiandrogen agents (abiraterone, enzalutamide, apalutamide, or darolutamide) or both.
- Mutation (germline or somatic) in the HRR pathway in either blood or biopsy samples.
- Written informed consent to participate.
- Eastern Cooperative Oncology Group performance status of 0-1.
- Aged older than 18 years.
- Adequate organ function:
  Absolute neutrophil count ≥1.5 × 109/L
  Hemoglobin ≥10.0 g/dL with no blood transfusions in the past 28 days.
  Platelet count ≥100 × 109/L.
  Total bilirubin ≤1.5 × institutional upper limit of normal (ULN).
  Aspartate aminotransferase (serum glutamic oxaloacetic transaminase) or alanine aminotransferase (serum glutamic pyruvate transaminase) ≤2.5 × institutional ULN unless liver metastases are present in which case, they must be ≤5 × ULN.
  Subjects must have an estimated creatinine clearance using the Cockcroft-Gault equation for men of ≥40 mL/minute.

Patients with any of the following will be excluded from the trial:

- Patients with symptomatic brain metastases. A scan to confirm the absence of brain metastases is unnecessary in all patients.
- Patients with spinal cord compression, unless considered to have received definitive treatment for this and evidence of clinically stable disease for 28 days.
- Patients are considered as poor medical risk due to a serious, uncontrolled medical disorder, nonmalignant systemic disease, or active, uncontrolled infection. Examples include but are not limited to uncontrolled ventricular arrhythmia, recent (within 3 months) myocardial infarction, uncontrolled major seizure disorder, unstable spinal cord compression, superior vena cava syndrome, extensive interstitial bilateral lung disease on high-resolution computed tomography scan, or any psychiatric disorder that prohibits obtaining informed consent.
- Patients with a known hypersensitivity to carboplatin.

One of this study’s investigators will obtain written informed consent from every patient before enrollment, during which the benefits and risks of participation will be informed in the local language (Hindi or English) that the patient comprehends. The study consists of two parts: (1) screening of tumor tissue or blood for mutations in the HRR pathway and (2) administration of carboplatin in patients found to harbor a deleterious or likely deleterious mutation in the HRR pathway.

Screening of mutations in the HRR pathway will be performed in the tumor tissue for the HRR genes using next-generation sequencing. The initial archived biopsy sample will be retrieved after informed consent is obtained. In patients where the initial biopsy sample is inaccessible or deemed to be of inadequate quality for next-generation sequencing, blood samples will be sent to assess germline mutations in the HRR pathway.

### Interventions

Patients who agree to participate in this study will be tested for the presence of known deleterious mutations in the archived tumor tissue in genes involved directly or indirectly in HRR pathway: *BRCA1*, *BRCA2*, *ATM*, *BRIP1*, *BARD1*, *CDK12*, *CHEK1*, *CHEK2*, *FANCL*, *MSH2*, *PALB2*, *PPP2R2A*, *RAD51B*, *RAD51C*, *RAD51D*, and *RAD54L*.

Those found to have a pathogenic or likely pathogenic mutation in one of these genes will be treated with 3 weekly carboplatin. The dose will be calculated according to the estimated glomerular filtration rate determined by the Cockcroft-Gault equation and then administered at the area under the curve (5) with a dose capped at 750 mg per cycle. Chemotherapy will continue until progression (clinical, biochemical, or radiographic) or intolerable adverse events, whichever occurs earlier. If the patient misses a follow-up visit, this study’s team will contact the patient and reinforce adherence to this study.

The relevant concomitant care permitted or prohibited during the trial includes the following. Palliative radiation for patients who develop urgent local complications in previously documented disease sites. The ideal gap between palliative radiotherapy and the first dose of carboplatin would be at least one week, and there would be no capping dose of palliative therapy.

Continuation of protocol therapy, if medically appropriate, should be discussed with the principal investigator at the site. The following medications and treatments are permitted in this study: (1) rescue medications for nausea or vomiting, (2) antibiotics, and (3) bisphosphonates or denosumab.

Certain medications may interact negatively with carboplatin or interfere with its effectiveness. The medications that may have significant interactions or should generally be avoided with carboplatin:

- Aminoglycoside antibiotics: these antibiotics, such as gentamicin or amikacin, may increase the risk of kidney damage when used concomitantly with carboplatin.
- Nonsteroidal anti-inflammatory drug: these drugs, including ibuprofen, naproxen, and aspirin, may increase the risk of bleeding or kidney damage when used concurrently with carboplatin.
- Live vaccines: live vaccines, such as the measles, mumps, rubella vaccine or varicella (chickenpox) vaccine, should generally be avoided during chemotherapy treatment, including carboplatin. These vaccines contain weakened live viruses that may pose a risk to immunocompromised individuals.
- Other nephrotoxic drugs: carboplatin can cause kidney toxicity, and combining it with other medications that have nephrotoxic effects, such as certain antibiotics or antifungal medications, may increase the risk of kidney damage.
- Anticoagulant medications: combining carboplatin with anticoagulants such as warfarin or heparin may increase the risk of bleeding. Close monitoring of blood clotting parameters is essential if these medications must be used together.
- Vaccines containing weakened or killed viruses or bacteria: while certain vaccines may be necessary for specific situations, it is important to discuss with a health care professional, as the timing and administration of vaccines should be carefully considered during chemotherapy treatment.

There are no medications or treatments that are specifically prohibited during this study.

All concomitant medications will be recorded at the time of registration.

Patients meeting either of the following criteria will discontinue this study’s treatment:

- Permanent discontinuation of carboplatin by treating physician (unacceptable toxicity or progression of underlying cancer).
- The investigator determines that continuing this study’s treatment is not in the patient’s best interest.
- Occurrence of an exclusion criterion affecting patient safety.
- Concomitant treatment that is not permitted.
- Failure to comply with the protocol. Suppose a patient consistently fails to attend scheduled assessments in this study. In that case, the investigator will determine the reasons and document the circumstances in the medical records as thoroughly and accurately as possible.
- The patient declines subsequent treatment or withdraws consent.

Posttrial care would be administered based on the treating physician’s discretion. Patients will be followed up until disease progression. Data collected for this study comprise clinical characteristics obtained from hospital records and routine laboratory investigations, including but not limited to baseline demographics, outcome measures, treatment details, and adverse events.

### Data Collection and Management

The research staff will collect data on site, using premade questionnaires on a paper case record form (CRF). All the parameters assessed in this study are defined a priori in a data dictionary, elucidating standards for data collection. Table 1 outlines the schedule of assessments.

Participating patients will be assessed before initiating a cycle of carboplatin-based chemotherapy and 3 weeks after the last dose of carboplatin. Subjects will be contacted by telephone if they miss their clinic appointment with the treating oncologist. Data entry will be done by using paper CRF. Data will be stored securely with limited access to authorized individuals. Biological samples will be collected as a part of this study at baseline. Formalin-fixed paraffin-embedded prostate tissue samples would be collected for HRR mutation testing. The patients’ blood samples would also be collected for germline testing of HRR mutations.

**Table 1:** Schedule of assessments.

	Screening	Baseline	Every cycle (3 weekly)	3 monthly
Informed consent	✓	—^a^	—	—
Serum testosterone	✓	—	—	—
Clinic assessment	—	✓	✓	—
Concomitant medications	—	✓	✓	—
Adverse events	—	—	✓	—
CBC^b^/LFT^c^/RFT^d^	—	✓	✓	—
Serum PSA^e^	—	✓	✓	—
Quality of life assessments	—	✓	✓	—
PSMA^f^ PET^g^ scan or CECT^h^ chest abdomen and bone scan	—	—	—	✓

^a^Not available.

^b^CBC: complete blood count.

^c^LFT: liver function test.

^d^RFT: renal function test.

^e^PSA: prostate-specific antigen.

^f^PSMA: prostate-specific membrane antigen.

^g^PET: positron emission tomography.

^h^CECT: contrast-enhanced computed tomography.

### Statistical Methods

The primary end point (confirmed PSA response rate) will be presented as a proportion to be tested against a 1-sided alternative.

Patient demographics, clinical and treatment characteristics, and other study outcomes will be described using mean, SD and range, or median, IQR and range for continuous variables, frequencies, and percentages for categorical variables, and the Kaplan‒Meier method for time-to-event variables. The effect sizes and 95% CIs will be presented where possible.

Simon’s [18] 2-stage design will be used. The null hypothesis that the true response rate (serum PSA decline of more than 50%) is 20% will be tested against a 1-sided alternative. In the first stage, 24 patients will be accrued. If there are 5 or fewer responses in these 24 patients, this study will be stopped. Otherwise, 25 additional patients will be accrued for a total of 49. The null hypothesis will be rejected if 15 or more responses are observed in 49 patients. This design yields a type I error rate of 5% and a power of 90% when the true response rate is 40%.

To include 49 patients with mCRPC harboring HRR mutations, we must screen approximately 200 patients, assuming a 30% prevalence and tissue inadequacy of 20%. The type of HRR mutation will analyze subgroups. Regression models (using the Cox proportional hazards model) will be used for exploratory analyses.

### Oversight and Monitoring

The principal investigator at the All India Institute of Medical Sciences, New Delhi, will coordinate this study and be responsible for data acquisition management and statistical analysis. Investigators from the Department of Medical Oncology at this study’s site will constitute the trial steering committee. The trial steering committee will evaluate any serious adverse events and report promptly to the Institute Ethics Sub Committee for Monitoring of Adverse Events in Clinical Trials at the site. Guideline-based collection of adverse event data will be ensured. Additionally, serious adverse reactions will be reported on time to the Institute Ethics Sub Committee for Monitoring of Adverse Events in Clinical Trials at the site. There will be no compensation offered for adverse events or serious adverse events to participants. After the initiation of this study, the trial steering committee will regularly audit the process of consenting, protocol adherence, and data collection biannually. Additionally, the data may be audited or inspected by the Institute Ethics Committee. Amendments made to the protocol will be communicated on time to the Institute Ethics Committee and the Clinical Trials Registry of India. One protocol amendment has been done in which we added the European Organisation for Research and Treatment of Cancer prostate cancer 25 questionnaire to the assessment and included the option of contrast-enhanced computed tomography chest abdomen and bone scan in place of a prostate-specific membrane antigen–positron emission tomography scan.

This trial will be published in a peer-reviewed journal with individual investigators as authors. Full credit will be given to the collaborating investigators and research staff involved in this study. All authors will review and approve this paper before submission and comply with internationally accepted requirements. We also plan to present this study’s results at major scientific meetings. The availability of results to all participants will be ensured.

### Ethical Considerations

The ethical approval has been obtained from the Institute Ethics Committee of the All India Institute of Medical Sciences, New Delhi who approved this study’s protocol on April 20, 2023 (IECPG-255/20.04.2023). Informed consent of each participant in the local language will be taken. The identifying information of participants will be removed as much as possible. Each participant will be given a study identification number, and the CRFs will contain only the deidentified data. Consent forms (with identifying patient data) will be safeguarded in locked compartments at this study’s site that can exclusively be accessed by authorized personnel. No monetary compensation will be provided to the enrolled participants. No identification of individual participants or users in any images of the paper or supplementary material is possible.

The protocol and final paper will be published as described by the International Committee of Medical Journal Editors guidelines. No professional writers will be employed to write the final paper. The supplementary material of the final paper will contain the full protocol. The deidentified data set and statistical code will be made available to the corresponding author at a reasonable request.

## Results

The trial started enrollment in September 2023. This trial is ongoing, and 12 patients have been recruited to date. All 49 participants will be enrolled according to plan.

## Discussion

### Relevance of the Study

This prospective phase II study will provide efficacy data of carboplatin in patients with mCRPC with deficient HRR. If this study provides positive results, it will provide a treatment option for patients with mCRPC. Further multicentric phase III noninferiority trials may be conducted to compare the efficacy with PARP inhibitors. Importantly, this study will provide a rationale for using carboplatin in resource-limited settings worldwide.

The limitation of this study is that it will have a screen failure rate of around 70%, as the prevalence of deleterious mutations in the HRR pathway is approximately 30%. Further, the availability of tumor tissue and the quality of archived specimens can also impair the technical ability to detect mutations in the HRR pathway. Therefore, approximately 200 patients with mCRPC will be screened to enroll 49 patients in this study.

There had only been small retrospective trials for using carboplatin in mCRPC. This would be the first phase II trial to evaluate the efficacy of carboplatin in patients with metastatic prostate cancer harboring mutations in the HRR pathway.

### Conclusions

This prospective phase II clinical trial addresses a critical unmet need in the management of patients with mCRPC harboring HRR gene mutations. With a focus on evaluating the efficacy of carboplatin, an affordable chemotherapy agent, this study holds promise as a new therapeutic option. The trial’s approach builds upon the concept of synthetic lethality, harnessed by both PARP inhibitors and carboplatin. If successful, carboplatin could emerge as a practical and accessible treatment strategy for this specific patient subset, potentially improving their outcomes and quality of life. By bridging the gap between high-cost therapies and broader patient accessibility, this study can potentially transform the landscape of mCRPC treatment, offering hope to patients worldwide.

## Appendix

Multimedia Appendix 1 Informed consent form.

Multimedia Appendix 2 Participant information sheet (PIS).

Multimedia Appendix 3 Participant informed consent form (PICF) Hindi.

## Abbreviations

CRF: case record form
HRR: homologous recombination repair
mCRPC: metastatic castration-resistant prostate cancer
PARP: polyadenosine 5’diphosphoribose (poly [ADP-ribose]) polymerase inhibitor
Poly (ADP-ribose): polyadenosine 5’diphosphoribose
PSA: prostate-specific antigen
ULN: upper limit of normal

## Data Availability

The supplementary material of the final paper will contain the full protocol. The deidentified data will be available upon reasonable request to the corresponding author after the publication of the final results.
