# Supplementary material for: Carboplatin in Patients With Metastatic Castration-Resistant Prostate Cancer Harboring Somatic or Germline Homologous Recombination Repair Gene Mutations: Phase II Single-Arm Trial
Source: JMIR Res Protoc. 2024 Apr 18;13:e54086. doi: 10.2196/54086 (PMC11066748; doi:10.2196/54086)
Supplement: Multimedia Appendix 1 [file resprot_v13i1e54086_app1.docx]

**Multimedia Appendix 1.** Informed consent form.

PARTICIPANT INFORMED CONSENT FORM (PICF)

Protocol/Study number:______________________

Participant identification number for this project: _______________________

Title of the Study/Project: “Carboplatin (AUC 5) in patients with metastatic castration-resistant prostate cancer harbouring somatic or germline homologous recombination repair (HRR) gene mutations: A phase II single-arm trial (CiPHeR)”

Principal Investigator

Dr. Rishabh Jain

Senior Resident

Department of Medical Oncology

Dr. B. R. A-IRCH,

All India Institute of Medical Sciences,

New Delhi-110029

Mobile: 7737133400

The contents of the information sheet that was provided have been read carefully by me/explained in detail to me in a language that I comprehend, and I have fully understood the contents. I confirm that I have had the opportunity to ask questions.

The nature and purpose of the study and its potential risks/benefits, the expected duration of the study, and other relevant details of the study have been explained to me in detail. I understand that my participation is voluntary and that I am free to withdraw at any time, without giving any reason, without my medical care or legal right being affected.

I understand that the information collected about me from my participation in this research and sections of any of my medical notes may be looked at by responsible individuals from AIIMS. I give permission for these individuals to have access to my records.

I agree to take part in the above study.

--------------------------------------------- Date:

(Signatures/Left Thumb Impression) Place:

Name of the Participant: ____________________________________

Son/Daughter/Spouse of:__________________________________

Complete postal address: _____________________________________

This is to certify that the above consent has been obtained in my presence.

------------------------------

Signatures of the Principal Investigator Date:

Place:

1) Witness – 1 2) Witness – 2

------------------------------ --------------------------------

Signatures Signatures

Name: Name:

Address: Address:
