# Supplementary material for: Carboplatin in Patients With Metastatic Castration-Resistant Prostate Cancer Harboring Somatic or Germline Homologous Recombination Repair Gene Mutations: Phase II Single-Arm Trial
Source: JMIR Res Protoc. 2024 Apr 18;13:e54086. doi: 10.2196/54086 (PMC11066748; doi:10.2196/54086)
Supplement: Multimedia Appendix 2 [file resprot_v13i1e54086_app2.docx]

**Multimedia Appendix 2.** Participant information sheet (PIS).

“Carboplatin in patients with metastatic castrate- resistant prostate cancer harboring somatic or germline homologous recombination repair (HRR) gene mutations: A phase II single-arm trial (CiPHeR)”

Principal Investigator

Dr. Rishabh Jain

Senior Resident

Department of Medical Oncology

Dr. B. R.A-IRCH,

All India Institute of Medical Sciences,

New Delhi-110029

Mobile: 7737133400

**1.** Aims and methods of the research:

Prostate cancer is one of the common cancers in men. When it spreads outside the prostate gland to other organs, like bone, liver, etc, it is known as metastatic prostate cancer. The initial treatment at diagnosis includes medical or surgical castration along with some other therapies. This stage of disease is called as metastatic castration sensitive prostate cancer. At a later stage, the disease starts progressing despite castration and this stage is known as metastatic castration-resistant prostate cancer (CRPC). Almost all patients with metastatic prostate cancer (mPC) go on to develop CRPC.

Around 25-30% of patients with mCRPC harbor some genetic alterations in the repair pathway of the genes, known as homologous recombination repair pathway (HRR). Somatic (in tumor biopsy) and germline (in blood) testing for HRR is validated and is widely available. In these selective patients, two poly ADP- ribose polymerase (PARP) inhibitors (olaparib and rucaparib) have shown an improvement in overall survival, and are approved as single agent drugs.

Interestingly, carboplatin is an inexpensive chemotherapeutic drug that also utilizes ‘synthetic lethality’ and is likely to be effective in this subgroup of patients with mCRPC. A recently published retrospective study showed an encouraging response rate of carboplatin in this setting. However, there are no prospective clinical trials that have looked into the role of carboplatin in patients with mCRPC harboring mutations in the HRR pathway. If effective, carboplatin may provide an inexpensive treatment option in resource-limited settings. Therefore, this phase II clinical trial is being conducted to evaluate the efficacy of carboplatin in patients with mCRPC harboring HRR gene mutations.

**2.** Expected duration of the subject participation:

At the first step, your tumor tissue will be checked for any mutation in the HRR genes (BRCA1, BRCA2, ATM, BRIP1, BARD1, CDK12, CHEK1, CHEK2, FANCL, PALB2, PPP2R2A, RAD51B, RAD51C, RAD51D, and RAD54L). If there are no mutations identified, you will not be able to participate further in the study.

If your test results show that one of the genes has a pathogenic mutation, you will be treated with carboplatin at a dose of AUC 5 administered over 2 hours every 3 weeks, calculated using your height, weight, and kidney function. This will be continued till your disease is responding and you do not develop any intolerable side effects due to carboplatin. Your routine blood tests will be

**3.** The benefits to be expected from the research to the subject or to the others: By participating in this study, you will contribute to the ongoing research. You will help advance what is known about the effect of carboplatin in prostate cancer.

**4.** Any risk to the subject associated with the study: Carboplatin is being used for several decades in oncology. Its side effects include infusion reactions, hypersensitivity, decrease in red blood cells, white blood cells, and platelets, generalized weakness, decreased appetite, peripheral neuropathy, febrile neutropenia, etc.

**5.** Maintenance of confidentiality of records: A code will be given to the patient and his name will not be disclosed All the information that you provide during the study will be kept confidential and will be utilized only for the study purpose.

**6.** Freedom of individual to participate and to withdraw from research at any time without penalty or loss of benefits to which the subject would otherwise be entitled: You are free not to permit the participation of yourself in the study. If you choose not to permit the participation of yourself, you will receive the usual available treatment and care.

**7.** Amount of blood sample to be taken should be mentioned in PIS in Tea Spoon full: 8 ml blood sample (one and a half tea spoonful) would be withdrawn at the time of enrollment for genetic mutation testing if the tumor tissue shows mutation in HRR genes.

**8.** Costs and source of investigations, disposables, implants and drugs/contrast media must be mentioned: The test (HRR and genetic testing) will be provided free of cost for you/your patient

**9.** It is important to understand the following points regarding genetic testing results:

a) Results from genetic testing may be positive, negative or inconclusive

º A positive result may confirm whether a person is affected with, a carrier of, or at risk for developing a genetic condition.

º A negative result does not exclude the possibility of being affected with or a carrier of a genetic condition. Genetic conditions may have many causes, some of which may not be completely known or testable.

º An inconclusive result may occur due to limitations of laboratory methods, limitations in knowledge of the meaning of identified variant(s), or poor sample quality. Inconclusive results from biochemical tests may occur due to an individual’s clinical status (fasting, illness, etc.) at the time the sample was drawn.

b) Identified genetic variants are interpreted using current information in the medical literature and scientific databases. Since this information can change, our laboratory may issue a revised report if the meaning of the variant changes. Individuals with a variant of uncertain significance should contact their healthcare provider periodically to determine if new information is available.

c) Genetic testing results may provide information that was not anticipated, such as:

º Identifying a genetic risk unrelated to the original reason for testing.

º Predicting another family member has, is at risk for, or is a carrier of a genetic condition.

º Revealing non‐paternity (the person stated to be the biological father is not, in fact, the biological father).

º Suggesting the parents of the individual tested are blood relatives.

d) Although genetic test results are usually accurate, several sources of error are possible, including clinical misdiagnosis of a condition, inaccurate information provided regarding family relationships, sample mis-labeling or contamination, transfusion, bone marrow transplantation, and maternal cell contamination of prenatal or cord blood samples

e) This study will submit HIPAA‐compliant, de‐identified (cannot be traced back to the patient) genetic test results and health information to public databases. The confidentiality of each sample is maintained.

f) Providing accurate information about symptoms and family history enables correct test selection and interpretation. In cases where a family member has tested positive for a genetic change, a copy of that report may be required by the laboratory before testing can be started.

**10.** Any time during the course of the study if you feel that you have not been adequately informed about the study or do not wish to continue in the study or have any query regarding the study, feel free to contact-

Dr. Rishabh Jain

Senior Resident

Department of Medical Oncology

Dr. B. R.A-IRCH,

All India Institute of Medical Sciences,

New Delhi-110029

Mobile: 7737133400
