# Supplementary material for: Carboplatin in Patients With Metastatic Castration-Resistant Prostate Cancer Harboring Somatic or Germline Homologous Recombination Repair Gene Mutations: Phase II Single-Arm Trial
Source: JMIR Res Protoc. 2024 Apr 18;13:e54086. doi: 10.2196/54086 (PMC11066748; doi:10.2196/54086)
Supplement: Multimedia Appendix 3 [file resprot_v13i1e54086_app3.docx]

**Multimedia Appendix 3.** Participant informed consent form (PICF) Hindi.

**प्रतिभागी सूचित सहमति प्रपत्र (PICF)**

प्रोटोकॉल / अध्ययन संख्या :______________________

इस परीक्षण के लिए प्रतिभागी पहचान संख्या: _______________

**अध्ययन / परियोजना का शीर्षक:** "मेटास्टेटिक कैस्ट्रेट प्रतिरोधी प्रोस्टेट कैंसर वाले रोगियों में कार्बोप्लाटिन, दैहिक या जर्मलाइन होमोलॉगसरिकॉम्बिनेशन रिपेयर (HRR) जीन म्यूटेशन को परेशान करता है: एक चरण II सिंगल-आर्म ट्रायल (CiPHeR)"

**डॉ. ऋषभ जैन**

**सीनियर रेसीडेंट**

**चिकित्सा ऑन्कोलॉजी विभाग**

**डॉ बी आर ए-आईआरसीएच,**

**अखिल भारतीय आयुर्विज्ञान संस्थान, नई दिल्ली-110029**

**फ़ोन-7737133400**

प्रदान की गई सूचना पत्र की सामग्री को मेरे द्वारा ध्यान से पढ़ा गया है / मुझे विस्तार से समझाया गया है, जिस भाषा में मैं समझता हूं, और मैंनेसामग्रीमैंने सामग्री को पूरी तरह से समझ लिया है। मैं पुष्टि करता हूं कि मुझे प्रश्न पूछने का अवसर मिला है।

अध्ययन की प्रकृति और उद्देश्य और इसके संभावित जोखिम / लाभ और अध्ययन की अपेक्षित अवधि, और अध्ययन के अन्य प्रासंगिक विवरण मुझेविस्तारमुझे विस्तार से बताए गए हैं। मैं समझता हूं कि मेरी भागीदारी स्वैच्छिक है और मैं बिना कोई कारण बताए, मेरी चिकित्सा देखभाल या कानूनी अधिकारकोअधिकार को प्रभावित किए बिना किसी भी समय वापस लेने के लिए स्वतंत्र हूं।

मैं समझता हूं कि इस शोध में मेरी भागीदारी से मेरे बारे में एकत्र की गई जानकारी और मेरे किसी भी मेडिकल नोट के अनुभागों को एम्स के जिम्मेदारव्यक्तियोंजिम्मेदार व्यक्तियों द्वारा देखा जा सकता है। मैं इन व्यक्तियों को अपने रिकॉर्ड तक पहुंच की अनुमति देता हूं।

मैं उपरोक्त अध्ययन में भाग लेने के लिए सहमत हूं।

---------------------------------------------

-------------------------------------------- दिनांक: स्थान:

(हस्ताक्षर/बाएं अंगूठे का निशान)

प्रतिभागी का नाम: ____________________________________

पुत्र/पुत्री/पति/पत्नी:__________________________________

डाक का पूरा पता: _____________________________

यह प्रमाणित किया जाता है कि उपरोक्त सहमति मेरी उपस्थिति में प्राप्त की गई है।

-----------------------------

प्रधान अन्वेषक के हस्ताक्षर दिनांक: जगह:

1) गवाह - 1 2) गवाह - 2

---------------------------- ------------------------ ------------------------

हस्ताक्षर हस्ताक्षर

नाम नाम

पता पता

नाम नाम

पता पता

 Appendix IV

 Patient information sheet **, Hindi**

**प्रतिभागी सूचना पत्र (पीआईएस)**

**अध्ययन / परियोजना का शीर्षक:** "मेटास्टेटिक कैस्ट्रेट प्रतिरोधी प्रोस्टेट कैंसर वाले रोगियों में कार्बोप्लाटिन, दैहिक या जर्मलाइन होमोलॉगस रिकॉम्बिनेशन रिपेयर (HRR) जीन म्यूटेशन को परेशान करता है: एक चरण II सिंगल-आर्म ट्रायल (CiPHeR)"

**अध्ययन / परियोजना का शीर्षक:** "मेटास्टेटिक कैस्ट्रेट प्रतिरोधी प्रोस्टेट कैंसर वाले रोगियों में कार्बोप्लाटिन, दैहिक या जर्मलाइन होमोलॉगस रिकॉम्बिनेशन रिपेयर (HRR) जीन म्यूटेशन को परेशान करता है: एक चरण II सिंगल-आर्म ट्रायल (CiPHeR)"

**1. अनुसंधान के उद्देश्य और तरीके:**

प्रोस्टेट कैंसर पुरुषों में होने वाले आम कैंसर में से एक है। जब यह प्रोस्टेट ग्रंथि के बाहर हड्डी, लीवर आदि जैसे अन्य अंगों में फैलता है, तो इसे मेटास्टेटिक प्रोस्टेट कैंसर के रूप में जाना जाता है। निदान के प्रारंभिक उपचार में कुछ अन्य उपचारों के साथ चिकित्सा या शल्य चिकित्सा बधियाकरण शामिल है। रोग के इस चरण को मेटास्टेटिक कैस्ट्रेशन सेंसिटिव प्रोस्टेट कैंसर कहा जाता है। बाद के चरण में, कैस्ट्रेशन के बावजूद रोग बढ़ना शुरू हो जाता है और इस चरण को मेटास्टेटिक कैस्ट्रेशन-प्रतिरोधी प्रोस्टेट कैंसर (सीआरपीसी) के रूप में जाना जाता है। मेटास्टेटिक प्रोस्टेट कैंसर (एमपीसी) वाले लगभग सभी रोगियों में सीआरपीसी विकसित होता है।

एमसीआरपीसी के लगभग 25-30% रोगियों में जीन के मरम्मत मार्ग में कुछ आनुवंशिक परिवर्तन होते हैं, जिन्हें समरूप पुनर्संयोजन मरम्मत मार्ग (एचआरआर) के रूप में जाना जाता है। एचआरआर के लिए सोमैटिक (ट्यूमर बायोप्सी में) और जर्मलाइन (रक्त में) परीक्षण मान्य है और व्यापक रूप से उपलब्ध है। इन चुनिंदा रोगियों में, दो पॉली एडीपी- राइबोज पोलीमरेज़ (PARP) अवरोधक (ओलापैरिब और रुकापैरिब) ने समग्र अस्तित्व में सुधार दिखाया है, और एकल एजेंट दवाओं के रूप में स्वीकृत हैं।

दिलचस्प बात यह है कि कार्बोप्लाटिन एक सस्ती कीमोथेरेपी दवा है जो 'सिंथेटिक घातकता' का भी उपयोग करती है और एमसीआरपीसी वाले रोगियों के इस उपसमूह में प्रभावी होने की संभावना है। हाल ही में प्रकाशित एक पूर्वव्यापी अध्ययन ने इस सेटिंग में कार्बोप्लाटिन की उत्साहजनक प्रतिक्रिया दर को दिखाया। हालांकि, यहां कोई संभावित नैदानिक ​​​​परीक्षण नहीं हैं, जिन्होंने एचआरआर मार्ग में एमसीआरपीसी के उत्परिवर्तन उत्परिवर्तन वाले रोगियों में कार्बोप्लाटिन की भूमिका को देखा है। यदि प्रभावी है, तो कार्बोप्लाटिन संसाधन सीमित सेटिंग्स में एक सस्ता उपचार विकल्प प्रदान कर सकता है। इसलिए, यह चरण II क्लिनिकल परीक्षण उन रोगियों में कार्बोप्लाटिन की प्रभावकारिता का मूल्यांकन करने के लिए आयोजित किया जा रहा है, जिनमें एचआरआर जीन उत्परिवर्तन एमसीआरपीसी है।

**2. विषय भागीदारी की अपेक्षित अवधि:**

पहले चरण में, एचआरआर जीन (बीआरसीए 1, बीआरसीए 2, एटीएम, बीआरआईपी 1, बार्ड 1, सीडीके 12, चेक 1, चेक 2, एफएएनसीएल, पीएएलबी 2, पीपीपी 2 आर 2 ए,) में किसी भी उत्परिवर्तन के लिए आपके ट्यूमर ऊतक की जांच की जाएगी। RAD51B, RAD51C, RAD51D और RAD54L)। यदि कोई उत्परिवर्तन नहीं पहचाना गया है, तो आप अध्ययन में आगे भाग नहीं ले पाएंगे।

यदि आपके परीक्षण के परिणाम दिखाते हैं कि जीन में से एक में रोगजनक उत्परिवर्तन है, तो आपको कार्बोप्लाटिन के साथ एयूसी 5 की खुराक पर हर 3 सप्ताह में 2 घंटे से अधिक प्रशासित किया जाएगा, जिसकी गणना आपकी ऊंचाई, वजन और गुर्दे के कार्य का उपयोग करके की जाएगी। यह तब तक जारी रहेगा जब तक आपकी बीमारी प्रतिक्रिया नहीं दे रही है और आप कार्बोप्लाटिन के कारण कोई असहनीय दुष्प्रभाव विकसित नहीं करते हैं। आपका नियमित रक्त परीक्षण होगा

**3. शोध से विषय या अन्य को होने वाले लाभ:**

इस अध्ययन में भाग लेकर, आप चल रहे शोध में योगदान देंगे। प्रोस्टेट कैंसर में कार्बोप्लाटिन के प्रभाव के बारे में जो ज्ञात है, उसे आप आगे बढ़ाने में मदद करेंगे।

**4. अध्ययन से जुड़े विषय के लिए कोई जोखिम:**

ऑन्कोलॉजी में कार्बोप्लाटिन का उपयोग कई दशकों से किया जा रहा है। इसके दुष्प्रभावों में जलसेक प्रतिक्रियाएं, अतिसंवेदनशीलता, लाल रक्त कोशिकाओं में कमी, सफेद रक्त कोशिकाओं और प्लेटलेट्स, सामान्यीकृत कमजोरी, भूख में कमी, परिधीय न्यूरोपैथी, ज्वर संबंधी न्यूट्रोपेनिया आदि शामिल हैं।

**5. अभिलेखों की गोपनीयता बनाए रखना:**

रोगी को एक कोड दिया जाएगा और उसके नाम का खुलासा नहीं किया जाएगा अध्ययन के दौरान आपके द्वारा प्रदान की जाने वाली सभी जानकारी को गोपनीय रखा जाएगा और केवल अध्ययन के उद्देश्य के लिए उपयोग किया जाएगा।

**6. व्यक्ति को बिना किसी दंड या लाभों के किसी भी समय अनुसंधान से भाग लेने और वापस लेने की स्वतंत्रता, जिसके लिए विषय अन्यथा हकदार होगा:**

आप अध्ययन में अपनी भागीदारी की अनुमति नहीं देने के लिए स्वतंत्र हैं। यदि आप स्वयं की भागीदारी की अनुमति नहीं देना चुनते हैं, तो आपको सामान्य रूप से उपलब्ध उपचार और देखभाल प्राप्त होगी।

**7. लिए जाने वाले रक्त के नमूने की मात्रा का उल्लेख पीआईएस में पूर्ण चाय के चम्मच में किया जाना चाहिए:**

8 मिलीलीटर रक्त का नमूना (डेढ़ चाय चम्मच) आनुवंशिक उत्परिवर्तन परीक्षण के लिए नामांकन के समय वापस ले लिया जाएगा यदि ट्यूमर ऊतक में उत्परिवर्तन दिखाता है एचआरआर जीन।

**8. जांच की लागत और स्रोत, डिस्पोजल, इम्प्लांट और ड्रग्स / कंट्रास्ट मीडिया का उल्लेख किया जाना चाहिए:**

परीक्षण (एचआरआर और आनुवंशिक परीक्षण) आपको / आपके रोगी के लिए मुफ्त प्रदान किया जाएगा।

**9. आनुवंशिक परीक्षण परिणामों के संबंध में निम्नलिखित बिंदुओं को समझना महत्वपूर्ण है:**

a) आनुवंशिक परीक्षण के परिणाम सकारात्मक, नकारात्मक या अनिर्णायक हो सकते हैं।

एक सकारात्मक परिणाम इस बात की पुष्टि कर सकता है कि क्या कोई व्यक्ति आनुवंशिक स्थिति विकसित करने के जोखिम में है, वाहक है या जोखिम में है।

एक नकारात्मक परिणाम आनुवंशिक स्थिति से प्रभावित या वाहक होने की संभावना को बाहर नहीं करता है। आनुवंशिक स्थितियों के कई कारण हो सकते हैं, जिनमें से कुछ पूरी तरह से ज्ञात या परीक्षण योग्य नहीं हो सकते हैं।

एक अनिर्णायक परिणाम प्रयोगशाला विधियों की सीमाओं, पहचाने गए प्रकार (ओं) के अर्थ के ज्ञान में सीमाओं, या खराब नमूना गुणवत्ता के कारण हो सकता है। नमूना लेने के समय किसी व्यक्ति की नैदानिक ​​स्थिति (उपवास, बीमारी, आदि) के कारण जैव रासायनिक परीक्षणों से अनिर्णायक परिणाम हो सकते हैं।

b) चिकित्सा साहित्य और वैज्ञानिक डेटाबेस में वर्तमान जानकारी का उपयोग करके पहचाने गए आनुवंशिक रूपों की व्याख्या की जाती है। चूंकि यह जानकारी बदल सकती है, हमारी प्रयोगशाला एक संशोधित रिपोर्ट जारी कर सकती है यदि संस्करण का अर्थ बदल जाता है। अनिश्चित महत्व के प्रकार वाले व्यक्तियों को यह निर्धारित करने के लिए समय-समय पर अपने स्वास्थ्य सेवा प्रदाता से संपर्क करना चाहिए कि क्या नई जानकारी उपलब्ध है।

c) आनुवंशिक परीक्षण के परिणाम ऐसी जानकारी प्रदान कर सकते हैं जो अपेक्षित नहीं थी, जैसे: परीक्षण के मूल कारण से असंबंधित आनुवंशिक जोखिम की पहचान करना।

परिवार के किसी अन्य सदस्य की भविष्यवाणी करना आनुवंशिक स्थिति के लिए जोखिम में है, या है।

गैर-पितृत्व प्रकट करना (जिस व्यक्ति को जैविक पिता कहा गया है, वह वास्तव में जैविक पिता नहीं है)।

सुझाव दिया गया है कि परीक्षण किए गए व्यक्ति के माता-पिता रक्त संबंधी हैं।

d) हालांकि आनुवंशिक परीक्षण के परिणाम आमतौर पर सटीक होते हैं, त्रुटि के कई स्रोत संभव हैं, जिनमें शामिल हैं: किसी स्थिति का नैदानिक ​​​​गलत निदान, पारिवारिक संबंधों के बारे में गलत जानकारी, नमूना गलत-लेबलिंग या संदूषण, आधान, अस्थि मज्जा प्रत्यारोपण, और मातृ कोशिका संदूषण। प्रसवपूर्व या गर्भनाल रक्त के नमूने।

e) यह अध्ययन सार्वजनिक डेटाबेस में एचआईपीएए-अनुपालन, गैर-पहचान (रोगी को वापस नहीं खोजा जा सकता) आनुवंशिक परीक्षण के परिणाम और स्वास्थ्य जानकारी प्रस्तुत करेगा। प्रत्येक नमूने की गोपनीयता बनाए रखी जाती है।

f) लक्षणों और पारिवारिक इतिहास के बारे में सटीक जानकारी प्रदान करना सही परीक्षण चयन और व्याख्या को सक्षम बनाता है। ऐसे मामलों में जहां परिवार के किसी सदस्य ने आनुवंशिक परिवर्तन के लिए सकारात्मक परीक्षण किया है, परीक्षण शुरू करने से पहले प्रयोगशाला द्वारा उस रिपोर्ट की एक प्रति की आवश्यकता हो सकती है।

10. अध्ययन के दौरान किसी भी समय यदि आपको लगता है कि आपको अध्ययन के बारे में पर्याप्त रूप से सूचित नहीं किया गया है या अध्ययन में जारी नहीं रहना चाहते हैं या अध्ययन के संबंध में कोई प्रश्न पूछना चाहते हैं, तो बेझिझक संपर्क करें-

**डॉ. ऋषभ जैन**

**सीनियर रेसीडेंट**

**चिकित्सा ऑन्कोलॉजी विभाग**

**कक्ष संख्या 160डी, प्रथम तल**

**डॉ बी आर ए-आईआरसीएच,**

**अखिल भारतीय आयुर्विज्ञान संस्थान, नई दिल्ली-110029**
